# The use of biologics in patients suffering from chronic rhinosinusitis with nasal polyps – a 4-year real life observation

**DOI:** 10.1007/s00405-024-08790-y

**Published:** 2024-07-08

**Authors:** Hanna Frankenberger, Robert Wiebringhaus, Benedikt Paul, Patrick Huber, Frank Haubner, Moritz Gröger, Clemens Stihl

**Affiliations:** 1 Department of Oto-Rhino-Laryngology, Head and Neck Surgery, Ludwig Maximilian University of Munich, Munich, Germany; 2 Department of Oto-Rhino-Laryngology, Head and Neck Surgery, Amper Hospital Dachau, Dachau, Germany

**Keywords:** CRSwNP, Biologics, Real-life effects, Type-2 inflammation, Long-term therapy course, Comorbidities

## Abstract

**Purpose:**

Antibody therapy for chronic rhinosinusitis with nasal polyps (CRSwNP) has been established in Germany since 2019. With limited long-term data on biologic treatment for CRSwNP, we conducted a comprehensive evaluation of our 4-year data. This monocentric study aims to assess the real-world effects of this treatment on clinical course, quality of life, treatment adherence, biologic switching, dual therapy, and comorbidities.

**Methods:**

We retrospectively analysed biologic therapy data in patients with severe chronic rhinosinusitis with nasal polyps. 191 patients with CRSwNP treated with Dupilumab, Mepolizumab, or Omalizumab were observed for up to 4 years in a real-life setting.

**Results:**

We observed clear symptom improvements with few side effects. No loss of efficacy or tolerability was noted during the 4-year period. Patients reported high satisfaction compared to previous therapies, with overall improved quality of life. Revision surgery or oral steroid use during biologic therapy was rare. Some patients prolonged injection intervals or discontinued steroid nasal spray. Biologic switching occurred infrequently due to side effects or inadequate response and was generally well tolerated. Many patients reported additional positive effects such as asthma or allergy symptom improvement and reduced medication intake.

**Conclusion:**

In summary, this study confirms the potency and tolerability of biologics for CRSwNP treatment, with sustained efficacy over 4 years. Biologic switching is a viable option for inadequate response or intolerable side effects. Therapy positively impacts Th2 comorbidities, corticosteroid requirements, surgery need, and overall compliance remains high.

**Clinical trial registration:**

Project No.: 22–0802. Registry name: Biologika bei Patient*innen mit chronischer Sinusitis mit Nasenpolypen.

## Introduction

Chronic rhinosinusitis (CRS) affects 3–5% of the population in Northern Europe, making it one of the most prevalent chronic inflammatory diseases with a steady increase in prevalence in recent years [1]. Incidence and endotype varies by geographic region [2], while the mean age of diagnosis ranges from 40 to 60 years [3]. Approximately 20–30% of CRS patients can be diagnosed with additional nasal polyps (CRSwNP).

CRSwNP is a complex multifactorial inflammatory disease of the nasal and paranasal mucosa frequently caused by underlying type 2 inflammation. Characteristic symptoms include impaired olfaction, nasal obstruction, facial pain, anterior or posterior rhinorrhoea, and occasionally headache. The resulting sleep and concentration dysfunction, and especially the olfactory disturbance, are often described as psychologically challenging [1, 4]. CRSwNP is associated with an increased incidence of depression and anxiety [5]. Radiologically and histologically, there is evidence of inflammatory changes in the sinonasal mucosa with hyperplastic tissue in the nasal cavities [6].

Inflammation in CRSwNP is characterized by type 2 CD4 + T helper cells (Th2) and infiltration of mucosa and polyp tissue with mast cells and eosinophilic granulocytes. In addition, the inflammatory response is characterized by elevated total IgE (Immunoglobulin E), IL (Interleukin) 4, -5, and − 13 in serum, plasma, and tissue [7]. Type 2 diseases typically affect organs with mucosal surfaces such as the respiratory tract, skin, or oesophagus. Many patients suffer from multiple type 2-mediated diseases during their lifetime, such as atopic dermatitis, bronchial asthma, eosinophilic esophagitis, aeroallergies and NERD (NSAID exacerbated respiratory disease) [1, 8]. Epidemiologic studies have shown that nearly half of patients with CRSwNP also have or will develop bronchial asthma and or allergic rhinitis in the future [8, 9].

The presence of such comorbidities as in Samter triad or Widal’s disease (the combination of aspirin intolerance syndrome and NSAID (Non-Steroidal Anti Inflammatory Drug(s))-induced asthma is associated with a more severe form of CRSwNP and is often more difficult to treat [10].

Until recently, the standard of care consisted of saline nasal irrigation and daily use of topical or, if symptom control was inadequate, systemic glucocorticosteroids. If symptom control could not be achieved by pharmacotherapeutic measures, functional endoscopic sinus surgery (FESS) was the treatment of choice [11]. Understanding the immunological basis of CRSwNP provides a highly effective alternative for patients with type 2 inflammation and a refractory, uncontrolled course through the use of monoclonal antibodies such as Dupilumab, Omalizumab or Mepolizumab.

Dupilumab was approved as the first monoclonal antibody-based drug for add-on maintenance therapy with nasal steroids in patients with CRSwNP in late 2019, followed by Omalizumab in 2020 and Mepolizumab shortly thereafter. These biologics are already established in the treatment of other type 2 inflammatory diseases. Dupilumab (Dupixent^®^) is a recombinant human monoclonal antibody that specifically binds to the IL-4Rα subunit of the common receptor of IL-4 and IL-13, thereby inhibiting both [7]. Omalizumab (Xolair^®^), another recombinant humanized monoclonal antibody, blocks IgE, which leads to a decreased activation of mast cells and basophilic granulocytes [12]. The most recently approved humanized monoclonal antibody is Mepolizumab (Nucala^**®**^). It binds to IL-5, thereby blocking its connection to the IL-5 receptor on eosinophil granulocytes [8]. The requirements for on-label biologic therapy in Germany, according to the S2k guideline, include severe uncontrolled disease, age over 18, and failure of established therapies. Additional criteria may include a Nasal Polyp Score (NPS) of more than 2 per side, unsuccessful use of oral steroids or Functional Endoscopic Sinus Surgery (FESS), Visual Analogue Scale (VAS) over 5, Sino-Nasal Outcome Test 2022 (SNOT22) over 40, and elevated eosinophil granulocytes.

We conducted a retrospective analysis of biological treatment for severe CRSwNP at our tertiary referral centre. Biological therapy, approved since 2019, is relatively new and costly. Despite comprehensive guidelines, uncertainties persist in its application. Treatment decisions are currently at the discretion of physicians. Little is known about long-term success, drug switching criteria, effects on comorbidities like asthma, Allergic Rhinitis (AR), or NERD, and long-term symptom improvement compared to each other. Therefore, this analysis aims to investigate real-life effects of different biological agents on clinical course, quality of life, compliance, therapy response, and comorbidities in routine healthcare in Germany.

## Methods

### Population and study design

This study is a monocentric observational study in a real-world clinical setting. 191 patients (119 males, 72 females; F:M = 1:1.6) with severe uncontrolled CRSwNP treated with subcutaneous Dupilumab, Omalizumab or Mepolizumab were enrolled. Patient excluded: one with a hearing and speech impairment, two with poor language skills, three who missed follow-up appointments and three who were missed by chance. Treatment took place between May 2019 and November 2023 at the ENT outpatient clinic of the LMU University Hospital in Munich, Germany. Approval for data analysis was granted by the LMU Ethics Committee in compliance with the Declaration of Helsinki § 15. Patients gave informed consent, and the clinical data was analysed pseudonymously. Therapy with biologics was carried out in accordance with the approval information of the individual drugs and the S2k guideline 2019/2023 of the German Society of Otorhinolaryngology, Head and Neck Surgery and the German Society of General Medicine [11]. European Forum for Research and Education in Allergy and airways disease (EUFOREA) modified supportive criteria for biologic therapy include recurrent polyps after FESS, the presence of bronchial asthma and evidence of type 2 inflammation [13].

Initial administration was supervised medically, with subsequent self-administration by patients. Quarterly check-ups were conducted at our clinic, every 3–4 months, where NPS, SNOT-22, olfactory tests, and VAS were reassessed. Treatment success was measured according to criteria outlined in the European Position Paper on Rhinosinusitis and Nasal Polyps (EPOS) by EUFOREA 2023, including improvements in NPS, SNOT-22, VAS, and olfactory test [13]. Furthermore, the treatment adherence, achievement of disease control in terms of need for surgery and/or oral corticosteroids and the effect of the therapy on other comorbidities and the resulting improved burden of disease were examined.

### Methodology and efficacy outcomes

The patients underwent nasal endoscopy and polyp scoring, a standardized smell test and self-assessment of the disease burden using the SNOT-22 questionnaire at both the initial examination and the clinical follow-up examination. The SNOT-22 score is a useful and widely used tool for quantifying quality of life and individual health burden in sinus diseases. Patients rate the severity of 22 symptoms on a six-point Likert scale, resulting in a total score from 0 to 110, with high scores indicating a high burden of rhinosinusitis-related health. The 22 questions are divided into four categories: rhinological symptoms, ear and facial symptoms, sleep function and psychological problems A questionnaire, including Visual Analogue Scale (VAS) assessments, was completed to track comorbidity and Quality of life (QoL) progression during therapy. The VAS is measured on a 100 mm scale, with 0 representing no burden and 100 mm the worst. Changes in asthma and AR symptoms, as well as medication reduction, were documented. Eosinophil counts and allergy screenings were conducted before therapy initiation, including allergy screening by skin prick test (LETI Pharma GmbH, Ismaning, Germany) and/or a serologic allergy screening panel (EUROIMMUN Medizinische Labordiagnostika AG, Lübeck, Germany). Nasal Polyp Score (NPS) was determined by endoscopy, ranging from 0 to 4, reflecting polyp severity [14].

Olfactory function was evaluated using either the B-SIT^®^ (Brief Smell Identification Test with 12 odors, Sensonics International) or the Sniffin’ Sticks Odor Identification Test (12 odor sets, Burghart Company, Wedel, Germany). These standardized tests present 12 odorants to identify from four possible responses in a multiple-choice format. Normal olfactory function is indicated by 11–12 correct identifications [15].

In addition, regular laboratory controls were performed during therapy to monitor eosinophil count and total IgE.

### Statistical analysis

Data were analysed using Matlab R2021b, Microsoft Excel, and PyCharm. Results were divided into quarters post-therapy initiation (defined as 91 days). Descriptive statistics determined means and standard deviations for symptom quantifications, comparing them to baseline significance using student’s t-test for normally distributed data (SNOT-22) and Mann-Whitney-U test for asymmetric distributions (NPS, Smelling Test), with significance level set at *P* < 0.05.

This method aimed to include a high sample proportion, avoiding exclusion due to precise follow-up dates. However, as patient follow-up decreased with treatment duration, sample sizes below 25 after four years were deemed unreliable for further analysis.

Polynomial regression (*n* = 5) approximated symptom decreases over time, excluding discrete data points. No subgroups were made according to individual biologics in the analysis.

## Results

### Baseline characteristics

At the time of data collection, 175 patients received 300 mg Dupilumab every two to four weeks, while 6 others were administered a weight-adjusted dose of Omalizumab every two or four weeks, and 8 patients received Mepolizumab every four weeks. Additionally, 4 patients underwent dual therapy with Dupilumab and Benralizumab due to severe uncontrolled asthma. The cohort had a mean age of 51.5 years. Among all patients, 75.3% reported comorbid asthma, 53.7% had allergic rhinitis, and 41.1% suffered from Samter triad. Furthermore, 67.9% had received oral steroid therapy at least once within a year before treatment initiation. Baseline parameters for each patient were obtained before antibody therapy was initiated, as detailed in Table 1.

**Table 1:** Baseline patient characteristics before biological therapy

Patient Characteristics		Baseline		*n*
Cohort Composition			SD / %	
	Age mean (SD)	51,49	13,17	191
	Female sex (%)	72	37,7	191
	Patients with IgE > 100 U/ml (%)	93	57,8	161
	Sensitization to aeroallergens (%)	98	74,8	131
Baseline Values			SD	
	Average VAS before therapy (0–10)	8,20	1,33	154
	Average SNOT-22 before therapy (0-110)	65,37	15,82	189
	Average NPS before therapy, right side (0–4)	2,66	0,95	188
	Average NPS before therapy, left side (0–4)	2,66	0,96	188
	Average Smelling Test before therapy (0–12)	4,36	5,36	186
	Average Eosinophilic Blood Count before therapy [G/l]	0,50	0,59	126
	Average IgE before therapy [U/ml]	252,48	350,09	161
Medical History			%	
	History of Asthma	143	75,26	190
	History of Allergy	102	53,68	190
	History of NERD	78	41,05	190

### Clinical outcome parameters

**Fig. 1:**
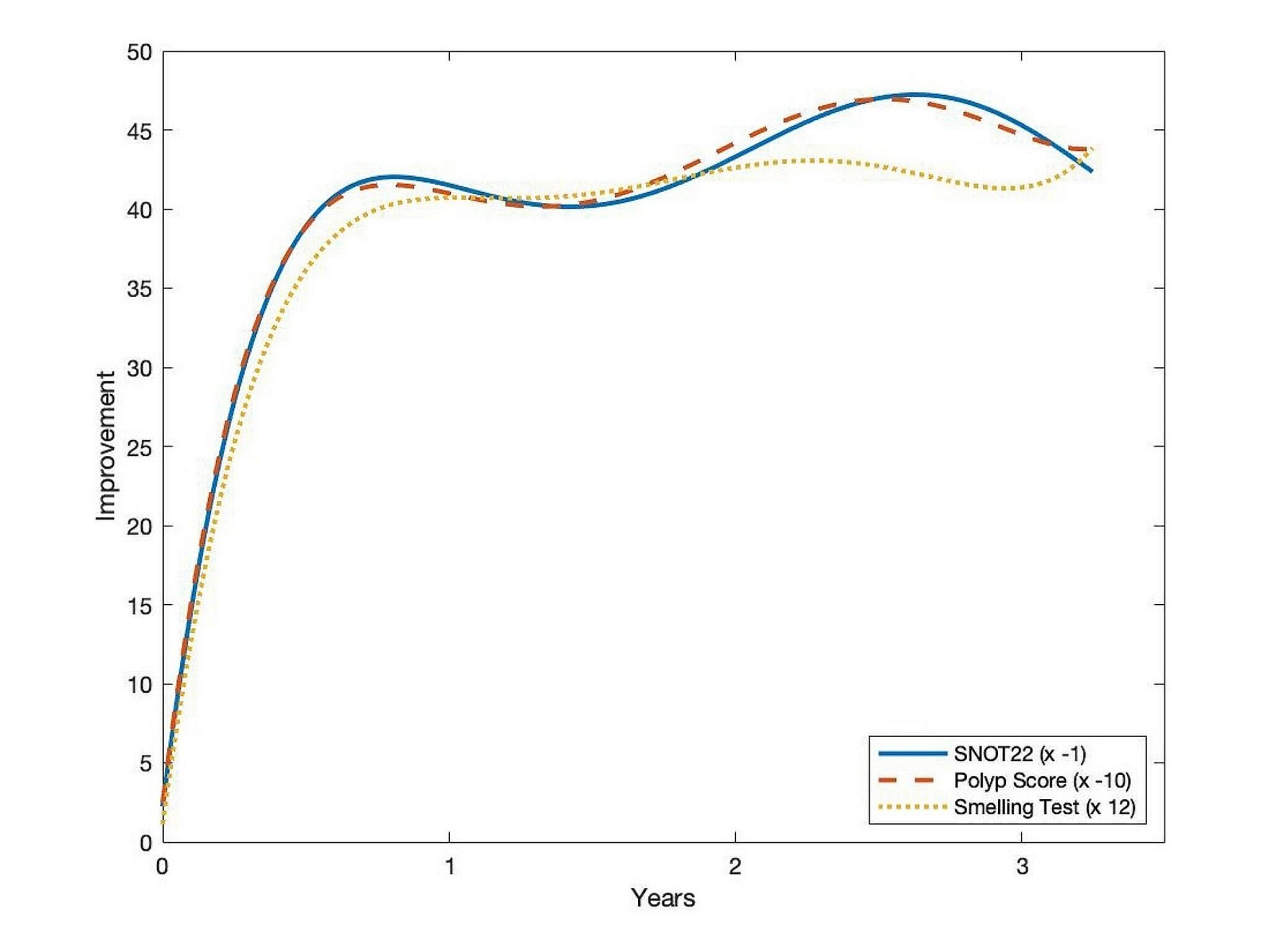
Development of NPS, SNOT-22 and olfactory test over time (polynomial regression with *n* = 5, R²= 0,95/0,94/0,92). In order to make the 3 parameters comparable, a scaling was used as shown in the box at the bottom right

There was a time synchronized improvement in polyp score, olfaction and quality of life as shown in Fig. 1.

**Fig. 2:**
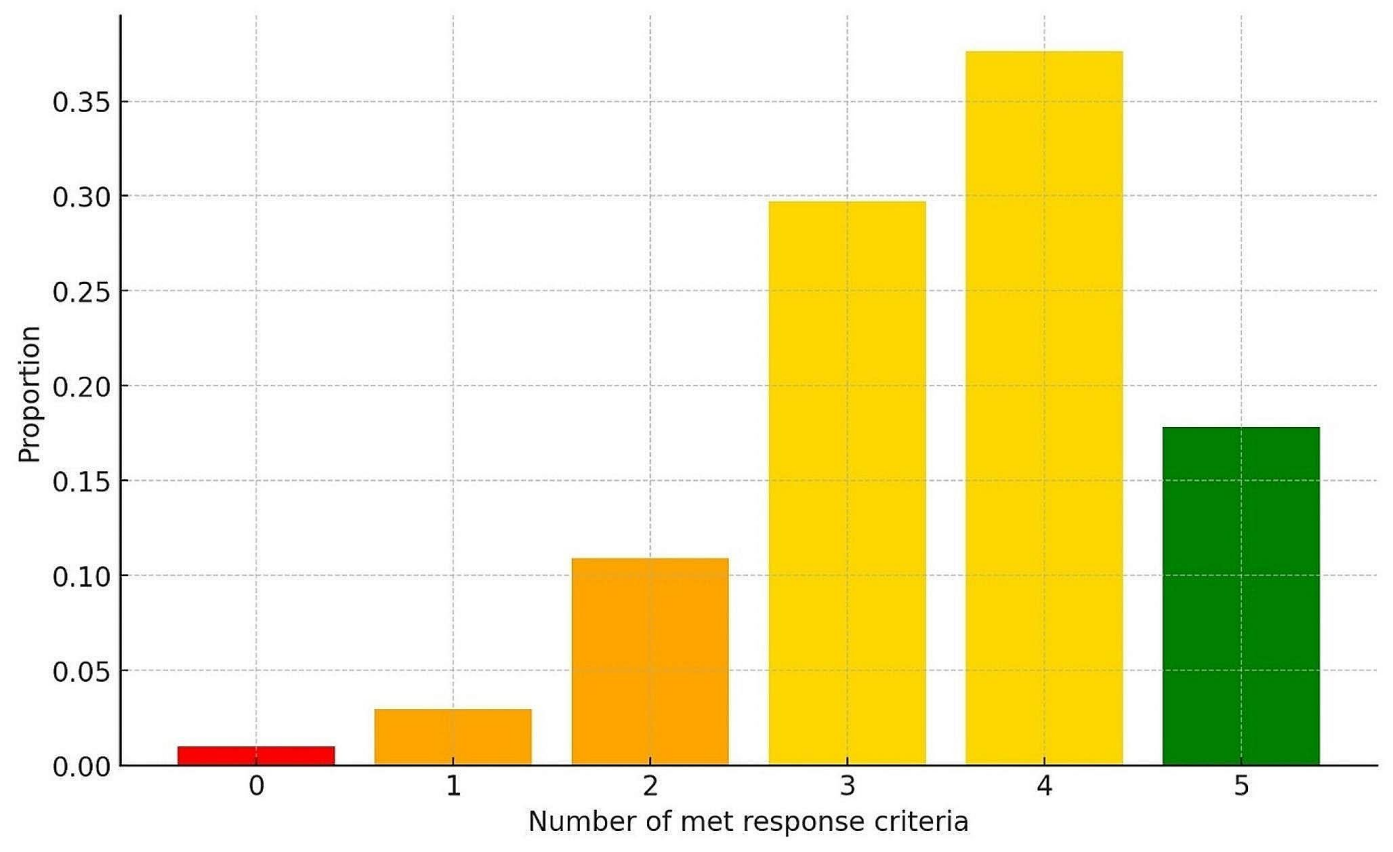
EUFOREA responder criteria 0–5 for biologics for patients with CRSwNP obtained 6 months after the start of therapy (Q1-2)

Response measured by NPS, SNOT-22, and smell improvement was generally satisfactory after 3 months per EUFOREA 2023 criteria, significantly differing (*p* < 0.001) from baseline averages after two years. At six months (Q1-Q2), responses were distributed as follows: super response (5 criteria) = 18%; moderate response (3–4 criteria) = 67.5%; poor response (1–2 criteria) = 13.5%; no response (0 criteria) = 1%. See Fig. 2 for distribution based on EUFOREA 0–5 response criteria.

6 criteria met = Super responder (green), 4–5 criteria met = moderate responder (yellow), 2–3 criteria met = Poor responder (orange), 0 criteria met = No responder (red).

**Fig. 3:**
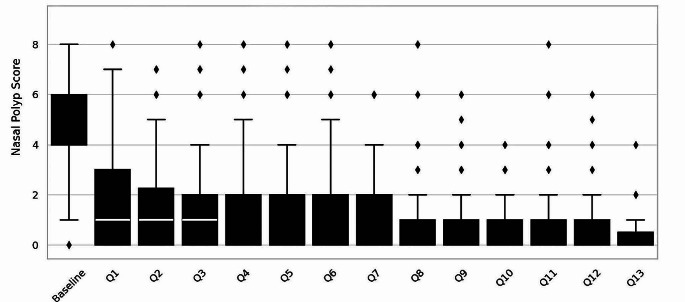
Progress of the nasal polyp score (0–4 per side, 0–8 both sides combined) under biological therapy measured quarterly (Q1, 2…) by rhinoscopy

After just one quarter (Q1), a rapid decrease in the nasal polyp score of initially 2.66 (SD ± 0.95) per side or 5.32 in total was observed. The NPS then continued to decrease over time and was still in a satisfactory range after 3 and a half years, as shown in Fig. 3.

After four years of therapy, there was no evidence of symptom control deterioration following an initial good response or increased side effects after good tolerability. Prior to treatment, patients had an average of 2.77 (SD ± 1.97) FESS procedures. During biologic therapy, 12 patients required re-FESS due to unsatisfactory polyp burden reduction. Before treatment, 129 patients reported using oral steroids at least once a year. During antibody treatment, 10 patients needed short-term systemic cortisone therapy due to inadequate CRSwNP symptom control, often during comorbidity exacerbations like allergic asthma.

### Quality of life during therapy

**Fig. 4:**
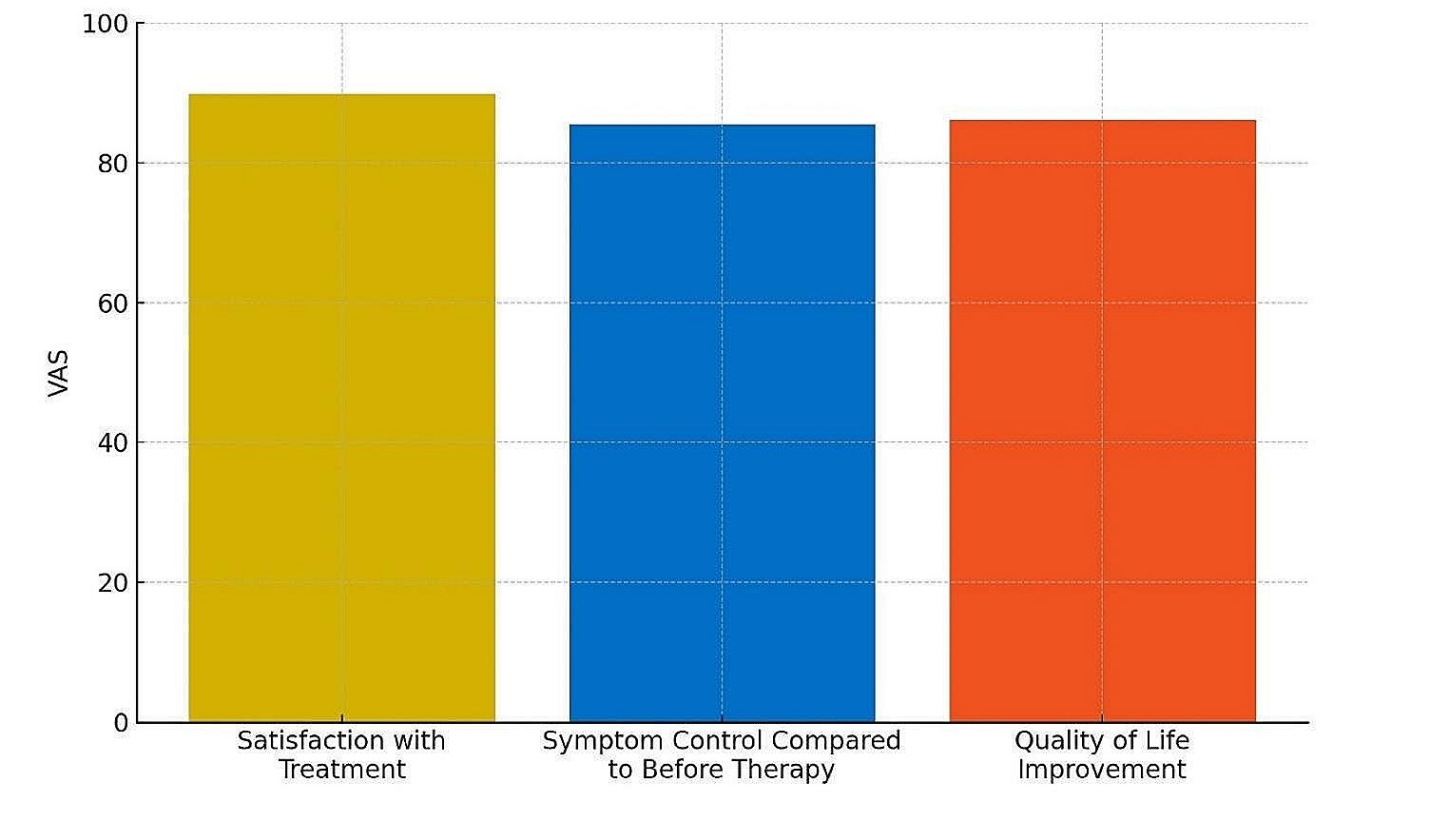
Improvement of treatment satisfaction, CRSwNP symptom control and QoL under biological therapy measured on a self-reporting VAS (0–100 mm)

Using a VAS scale (0 = very dissatisfied, 10 points/100 mm = maximally satisfied), patients rated satisfaction with their current treatment at 89.7/100 mm compared to before antibody therapy (Fig. 4). Symptom control under antibody therapy was rated at 85.4/100 mm on the VAS. Quality of life improvement during therapy was reported as 86.0/100 mm on the VAS.

### Comorbidities during therapy

From the observed patient population, 143 patients (75,3%) were reported to suffer from comorbid bronchial asthma. Of these, 92 (64,3%) reported a subjectively clear improvement of more than 75/100 mm in their asthmatic symptoms on the VAS. 106 of these 143 patients reported a reduction in the dosage or frequency of their asthma-specific medication and/or long-term therapy.

Allergic rhinitis was another commonly seen comorbidity. As shown in Tables 1 and 98 patients were tested positive for sensitization to at least one allergen in the skin prick test (SPT) or immunoblot. SPT was performed only in cases of suspected sensitization. Of the observed patient population, 102 patients self-reported comorbid allergic rhinitis (53,7%). Of these, 38 (37,25%) showed a clear improvement of more than 75/100 mm in their allergic symptoms on the VAS (VAS 0 = no improvement, 10 = best improvement imaginable). 36 of these 102 patients reported a reduction in the dosage or frequency of their anti-allergic medication (35,3%).

At baseline, 93 of 191 patients had elevated blood IgE levels above 100 UE/ml. A general decrease in total IgE compared to the baseline value was also observed over the course of the observation. The mean baseline IgE measured 252,5 UE/ml. The IgE values after two years differed significantly by 88,0 UE/ml (*P* < 0,001) to the baseline average.

### Side effects and switching

35 of the 191 patients (18.3%) reported side effects, the most common complaint being redness and pain at the injection site, conjunctival irritation followed by fever and pruritus. Of these, 12 of the 191 patients reported that the side effects were intolerable, resulting in a change in therapy.

A total of 27 patients were switched from one agent to another after 367 days on average. This was equally due to intolerable side effects or non-response. The most common switch was from Dupilumab to Mepolizumab (9 patients). Less frequently, patients were switched from Dupilumab to Omalizumab (3 patients) or from initial Omalizumab to Dupilumab (3 patients). In a few cases, patients were switched from Omalizumab to Mepolizumab or vice versa.

However, 4 patients were switched back to the original preparation because the clinical course did not develop as anticipated. The switch was generally well tolerated and usually carried out without a wash-out period.

4 patients received dual therapy with Dupilumab and Benralizumab, an anti-IL5Rα antibody. This was indicated in all patients due to severe refractory asthma by pulmonology. The dual therapy was well tolerated in all cases and led to a significant improvement in CRSwNP and reported satisfactory asthma improvement. No increased side effects were observed.

### Therapy adherence

It was also observed that some patients used their ICS nasal spray infrequently (47/191 = 24.6% patients) or stopped using it altogether (19/191 = 10%), despite being informed that taking INC is mandatory due to the add-on character of biologic therapy.

During the observation period, 74 of 191 patients (38,7%) reported that they had self-initiated an extension of their injection interval, despite being informed of the regulatory requirements. Of the patients who initially received Dupilumab every 2 weeks, 27 patients extended the injection interval to every 3 weeks and 30 patients to every 4 weeks.

## Discussion

The efficacy and safety of biologics as a therapy for CRSwNP has been largely confirmed [4, 12, 13, 15]. However, according to the EPOS/EUFOREA update of June 2023 [13] there is still a lack of data on long-term effectiveness and use of biologics in CRSwNP in a real-world setting. Furthermore, there are only few studies describing the switching from one biologic to another [16, 17] or dual therapy approaches. Most available literature on non-standard treatment modalities revolve merely around the extension of injection intervals [18]. We aimed to further investigate these questions during our 4-year analysis in addition to collecting the regular follow-up parameters. Throughout the study period, special attention was paid to comorbidities and QoL.

In this relatively large cohort of 191 patients followed for up to of 4 years, a significant symptom improvement was observed in a real-world setting with few side effects that were well tolerated by most patients. Our baseline and key clinical outcome parameters are consistent with other epidemiologic studies [9] and phase 3 trial studies, such as the LIBERTY NP SINUS-24 and LIBERTY NP SINUS-52 [19]. The congruence with well-conducted studies underscores the power of the present data analysis.

There was no decrease in efficacy or tolerability during the observation period of nearly 4 years, which is demonstrated in Fig. 1. We observed a dramatic time-synchronized improvement in NPS, olfactory function and SNOT-22 congruent to the findings of other studies [15, 19]. With decreasing NPS, shown in Fig. 3, a simultaneous improvement in olfactory function was seen, suggesting a primarily obstruction related and Th2 inflammatory cause of the olfactory disorder rather than lasting destruction of epithelia. The loss of smell in CRSwNP is not yet fully understood and needs to be further investigated [20].

The clinical improvement was mainly observed during the first year, which aligns with previous findings [21]. Interestingly, continued improvements in olfaction, SNOT22 and NPS were observed beyond the first year, as shown in Fig. 1. However, due to the small number of patients reaching the 4-year endpoint, the exact extent of improvement could not be quantified. Further analysis is ongoing. The favourable response to biologic therapy led more than one-third of patients to self-extend injection intervals and more than one-fifth to irregularly use or discontinue their ICS nasal spray. This self-extension may explain the slight dip in the curve after one year in Fig. 1. In 57 /190 patients, satisfactory therapeutic success was observed despite extended injection intervals. This could indicate that extended injection intervals may be sufficient for a certain patient group with initially very good response. It’s important to note that not all patients attempted to extend their injection interval.

During ongoing therapy, the corticosteroid-containing nasal spray was used less consistently or not at all, especially by patients who experienced dry nasal mucosa or nosebleeds, which are commonly reported side effects [22]. This had no visible effect on the NPS.

The therapy also led to a significant decrease in oral steroid use and the need for FESS surgery which has been described in other publications [23, 24]. We can now confirm this over the course of 4 years. Patients who do not want to or cannot undergo another surgery are therefore usually well treated with biologics. It should be noted, however, that the treatment of CRSwNP with FESS is significantly more cost-effective than treatment with biologics [25]. However, it is associated with more surgery-related complications [26].

Asthma and AR were common comorbidities [1, 10, 27], and many patients reported improvement in asthma (64.3%) and/or AR-related symptoms (37.2%) and were able to reduce their medication regimen, demonstrating the significant health benefits of biologic therapy in patients with multiple Th2 inflammatory diseases. This reduction in medication use can minimize polypharmacy and adverse side effects.

In addition to significant improvements in SNOT22, NPS and olfactory function, our questionnaire revealed high levels of satisfaction compared to previous therapeutic options. Overall quality of life and VAS scores also improved, consistent with findings from other studies [24, 28]. The positive health effects appear to be significant enough to outweigh the burden of regular self-injection and medical appointments, so that satisfaction and compliance remained high even after several years.

In an interdisciplinary approach, 4 patients were treated with a dual therapy of Dupilumab for CRSwNP by ENT and Benralizumab for severe persistent asthma by pulmonology. Despite the now proven efficacy of Benralizumab for CRSwNP [29], it did not lead to a satisfactory improvement in nasal symptoms in these 4 patients. Thus, the decision was made to add Dupilumab. This resulted in the desired improvement in nasal symptoms with stable asthma control and without additional side effects. Data on dual antibody therapy with Dupilumab and Benralizumab are not yet available. Further studies are required.

Switching between biologics occurred because of intolerable side effects or inadequate response to initial therapy, usually without a wash-out period to prevent clinical deterioration. Although generally well tolerated, this approach should be discussed with patients beforehand to avoid unnecessary switching back.

Limitations of this study include that it was conducted at a single centre at LMU University Hospital, which may have favoured patients with severe CRSwNP. Data density decreased over time as there were fewer patients towards the end of the 4-year observational period. However, the sample size remained large enough to assume a normal distribution. Follow-up visits varied in frequency based on medication and injection interval.

Most patients received Dupilumab, which is considered to be superior to other biologics in studies and meta-analyses [30–32]. Only a few patients currently receive Mepolizumab or Omalizumab, making statistical comparison between agents impossible. It’s important to note that despite all three biologics reducing CRSwNP symptoms, they vary in mechanism and effects on clinical parameters. This real-world study included all patients, regardless of the specific biologic used or changes made. The aim was to demonstrate the general benefits of biological treatment, focusing on symptom burden and validated questionnaires rather than laboratory parameters or histology.

Future research will be necessary regarding the long-term effects of therapy beyond 4 years and to conduct double-blind standardized trials comparing Dupilumab, Omalizumab, and Mepolizumab. This will help determine which patient subpopulation benefits most from each agent. Additionally, off-label interval prolongation in patients with initially satisfactory outcomes warrants further investigation.

In summary, this study confirms that biologics are potent and well-tolerated treatments for CRSwNP, with no expected decrease in efficacy or tolerability over 4 years. Switching between agents was well-tolerated, and therapy positively impacted Th2 comorbidities, corticosteroid requirements, need for surgery, quality of life, and overall compliance.

## Data Availability

not applicable.
